# Long term impact of child abuse in university students

**DOI:** 10.1186/s12888-026-08182-y

**Published:** 2026-05-25

**Authors:** Amir Soliman, Reem Hamdy, Amira H. Mohammed

**Affiliations:** 1 Department of Public Health and Community Medicine, Faculty of Medicine, Delta University for Science and Technology, Gamasa, Egypt; 2 Faculty of Medicine, Delta University for Science and Technology, Gamasa, Egypt; 3 Department of Physical Therapy for Pediatrics, Faculty of Physical Therapy, Delta University for Science and Technology, Gamasa, Egypt

**Keywords:** Long term impact, Child abuse, University students

## Abstract

**Background:**

A growing body of research underscores the detrimental impact of child maltreatment on a child’s development. Encompassing a wide range of actions by caregivers that significantly impede optimal development and violate societal norms, child maltreatment can manifest in various forms, including neglect of physical, cognitive, emotional, and social needs. Accordingly, this study aimed to describe the self-reported psychological, social, and physical sequelae reported by Egyptian university students with histories of childhood maltreatment.

**Methods:**

Employing a cross-sectional design, researchers conducted the study over a six-month period. Participants completed self-report questionnaires in the presence of research team members. The questionnaires were designed to assess the type of child abuse experienced, along with its long-term physical, social, and psycho-behavioral effects.

**Results:**

The findings describe a high prevalence of self-reported psycho-behavioral and physical health difficulties among university students with histories of childhood maltreatment. Specifically, participants reported experiencing symptoms of generalized anxiety, low self-esteem, feelings of inadequacy, and post-traumatic stress symptoms. Notably, gastrointestinal problems emerged as the primary physical health concern reported among participants with histories of childhood maltreatment.

**Conclusion:**

This study describes a substantial burden of self-reported psychological and physical difficulties among university students with histories of childhood maltreatment. Because the study was descriptive, cross-sectional, and based on a non-validated self-report questionnaire, further research using validated measures and more rigorous designs is needed.

**Trial registration:**

The study was retrospectively registered with ClinicalTrials.gov under the identifier NCT06366126 on April 10, 2024.

**Supplementary Information:**

The online version contains supplementary material available at 10.1186/s12888-026-08182-y.

## Background

 Childhood maltreatment, encompassing physical, emotional, sexual abuse, and neglect, is a pervasive public health concern with lasting consequences [1]. While the immediate effects of abuse are undeniable, the long-term impacts cast a long shadow, shaping an individual’s physical and mental health, social interactions, and overall well-being throughout their life [2]. This study explores the long-term effects of child abuse, highlighting the diverse ways it influences a survivor’s life trajectory.

A growing body of research from the past two decades underscores the enduring detrimental effects of child abuse. Studies consistently report associations between childhood maltreatment and negative outcomes in adulthood, encompassing physical, psychological, behavioral, and social domains [3, 4].

Individuals with a history of child abuse exhibit an increased risk of developing chronic health problems later in life. The chronic stress response triggered by abuse disrupts brain and nervous system development, potentially leading to an increased susceptibility to physical conditions like cardiovascular disease, respiratory issues, and diabetes [3, 5].

Survivors of child abuse are disproportionately represented among populations struggling with mental health disorders. Studies consistently report a higher prevalence of depression, anxiety, post-traumatic stress–related symptoms, and substance abuse in this population [4, 6]. These conditions can significantly impair an individual’s ability to function in daily life.

Children who have been abused often exhibit behavioral problems like aggression, delinquency, and self-harm [7, 8]. These difficulties can lead to academic struggles, strained relationships, and legal problems.

Abuse experiences can negatively impact a child’s capacity to form healthy relationships. Survivors may struggle with trust, maintain unhealthy attachments, and have trouble establishing intimacy [9, 10]. This can lead to social isolation and feelings of loneliness. The severity and duration of the abuse, the child’s age at the time of the abuse, and the availability of supportive relationships are crucial factors influencing the long-term consequences [3, 8]. However, the research consistently highlights the significant and often lifelong impact of child abuse on survivors. A substantial body of researches underscores the detrimental long-term effects of child abuse across physical, psychological, behavioral, and social domains. Understanding these consequences is paramount for developing effective prevention and intervention strategies to support survivors and ultimately break the cycle of abuse.

Child abuse in Egypt and the MENA region is an important public health problem but is not being discussed thoroughly enough. Child abuse is underreported for several reasons, including stigma, social stigma, the social pressure on victims, and the issue of reporting. Furthermore, there are several barriers that prevent victims of childhood abuse from accessing mental health services; these barriers are particularly prevalent among adolescents and young adults and can delay an individual’s ability to recognize and/or seek help for psychological distress caused by experiencing abuse. In Egypt, very little research has been done on the long-term impact of childhood maltreatment, despite the need for such research. Cultural and systemic factors may impact the behaviorality of reporting and the long-term recovery of individuals who have experienced maltreatment, indicating a need for more culturally specific research. As such, this study aimed to describe the self-reported psychological, social, and physical effects of childhood maltreatment among Egyptian university students.

## Methods

### Study design

This study employed a cross-sectional design, in which data were collected at a single point in time directly from participants using self-report questionnaires during the study period. The study did not involve secondary data analysis or the use of existing medical, academic, or administrative records. This cross-sectional study, adhering to the ethical principles of the Declaration of Helsinki (1975), was conducted in 2023 to safeguard participant welfare. Though retrospective in data collection, the study was subsequently registered with ClinicalTrials.gov (NCT06366126) on April 10, 2024. Prior to commencement, the Faculty of Medicine, Al-Azhar University, Damietta’s ethics committee granted approval (Number: DFM-IRB 00012367-22-02-008). Informed consent was obtained from all participants following a comprehensive explanation of the study’s objectives and design. Participant confidentiality and the right to withdraw from the study at any point were explicitly emphasized.

### Participants

A sample of 242 university students (91 males, 151 females) was recruited from Al Dakahlia governorate, Egypt. All participants had self-reported histories of childhood maltreatment identified through structured questionnaires administered by the research team. The term ‘documented’ refers to systematic questionnaire-based documentation and does not imply legal records, judicial involvement, or prior clinical diagnosis. Histories of childhood maltreatment were based solely on retrospective self-report and were not confirmed using structured interviews, legal records, or clinical documentation.

Sample size estimation was guided by prior descriptive studies of similar populations and by feasibility considerations; the achieved sample size was considered sufficient to provide stable descriptive estimates. The study adopted stringent inclusion criteria. Participants were recruited from multiple universities located in Al Dakahlia governorate, Egypt, using a convenience sampling approach through general student outreach during the academic term. This convenience sampling approach may have introduced selection bias and limits the generalizability of the findings to the broader university student population. Participation was voluntary, and all students provided written informed consent prior to enrollment in the study. Individuals lacking confirmed histories of child abuse were excluded from the study.

### Outcome measures

A tailored questionnaire designed to collect socio-demographic data and history related to abuse and its sequelae (Appendix I). It used to evaluate type and physical, social and psycho behavioral long-term impact of child abuse.

The study utilized a structured self-report questionnaire to collect data on long-term effects of child maltreatment. The questionnaire measured several domains including psychological (symptoms of anxiety, trauma-related distress, low self-esteem, obsessive thoughts), social (behavioral, legal, interpersonal), and physical health outcomes (gastrointestinal complaints, somatic symptoms). Items were formulated as non-diagnostic questions capturing participants’ subjective perceptions and experiences rather than clinically confirmed conditions. The results were analyzed descriptively; there were no diagnostic cut-offs used. The analysis also treated every response item as an indicator of whether the participant self-reported the condition/experience. No composite scores nor diagnostic cut-off scores were used. The interpretation of the findings reflects self-reporting of mental and/or physical health status and not based on clinically established diagnoses. Because the questionnaire was developed for this study and was not psychometrically validated, the findings should be interpreted as descriptive self-reported perceptions rather than standardized measurements of clinical outcomes.

Psychological and physical health outcomes, including symptoms consistent with post-traumatic stress, obsessive–compulsive behaviors, and eating-related difficulties, were assessed using self-report questionnaire items. Participants were asked whether they had ever experienced or perceived these conditions. No validated diagnostic instruments were used, and no clinical or psychiatric diagnoses were confirmed by health professionals. Therefore, reported outcomes reflect self-perceived mental and physical health conditions rather than formally diagnosed with psychiatric disorders.

### Statistical analysis

The analysis focused exclusively on descriptive statistics. Continuous variables were summarized using means and standard deviations or medians and interquartile ranges, as appropriate. Categorical variables were summarized using frequencies and percentages. No inferential statistical tests, subgroup comparisons, hypothesis testing, or multivariable analyses were performed; accordingly, the study was not designed to test associations, identify predictors, or estimate independent effects.

## Results

### Participant characteristics

Table 1 summarizes participant demographics. The study included 242 participants, with females constituting roughly two-thirds (66.7%) and males the remainder. The average participant age was 20.07 years old (SD = 1.14). Regarding residence, most participants (83.3%) resided in rural areas.

### Social consequences of childhood maltreatment

**Fig. 1:**
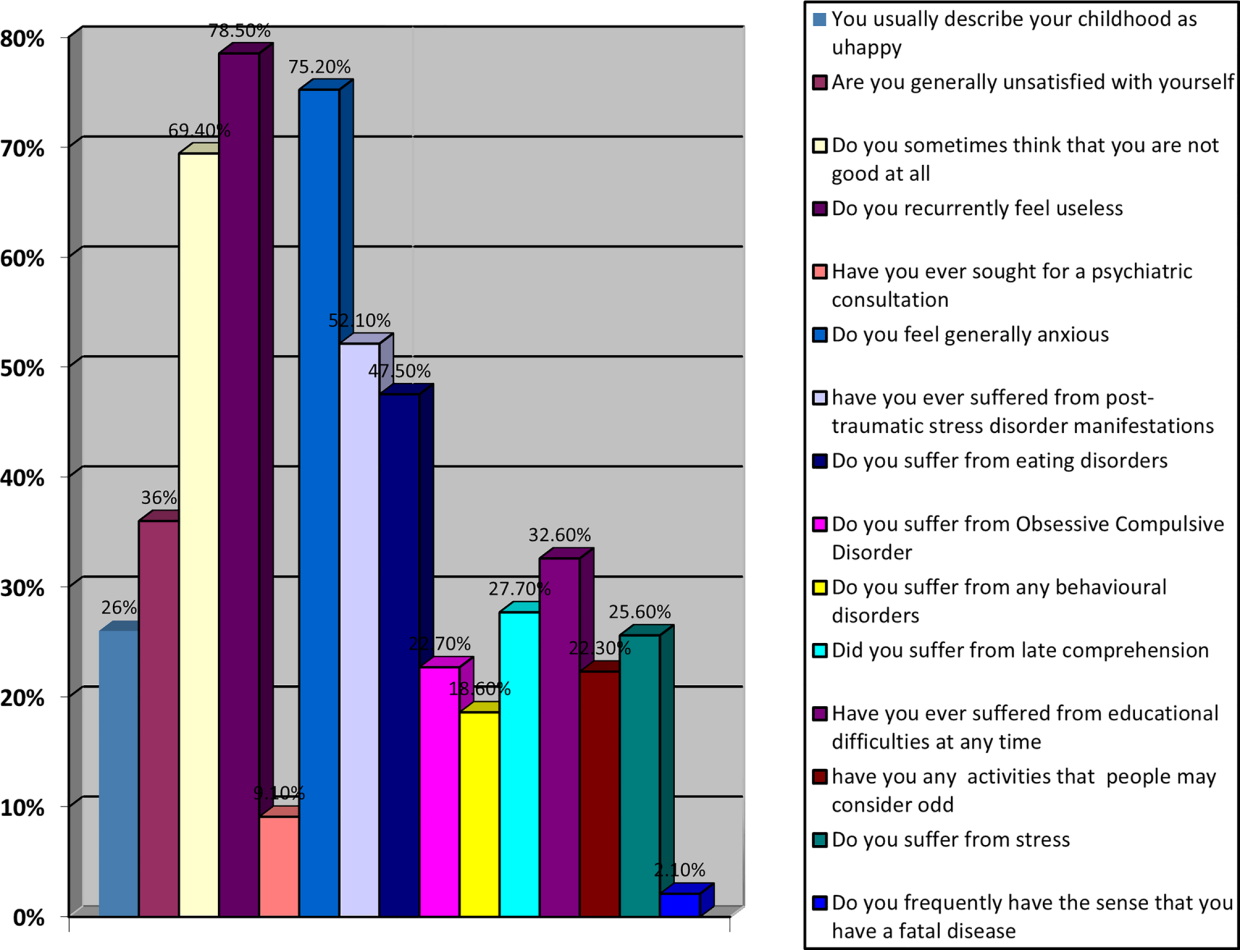
Psycho-behavioral consequences

**Fig. 2:**
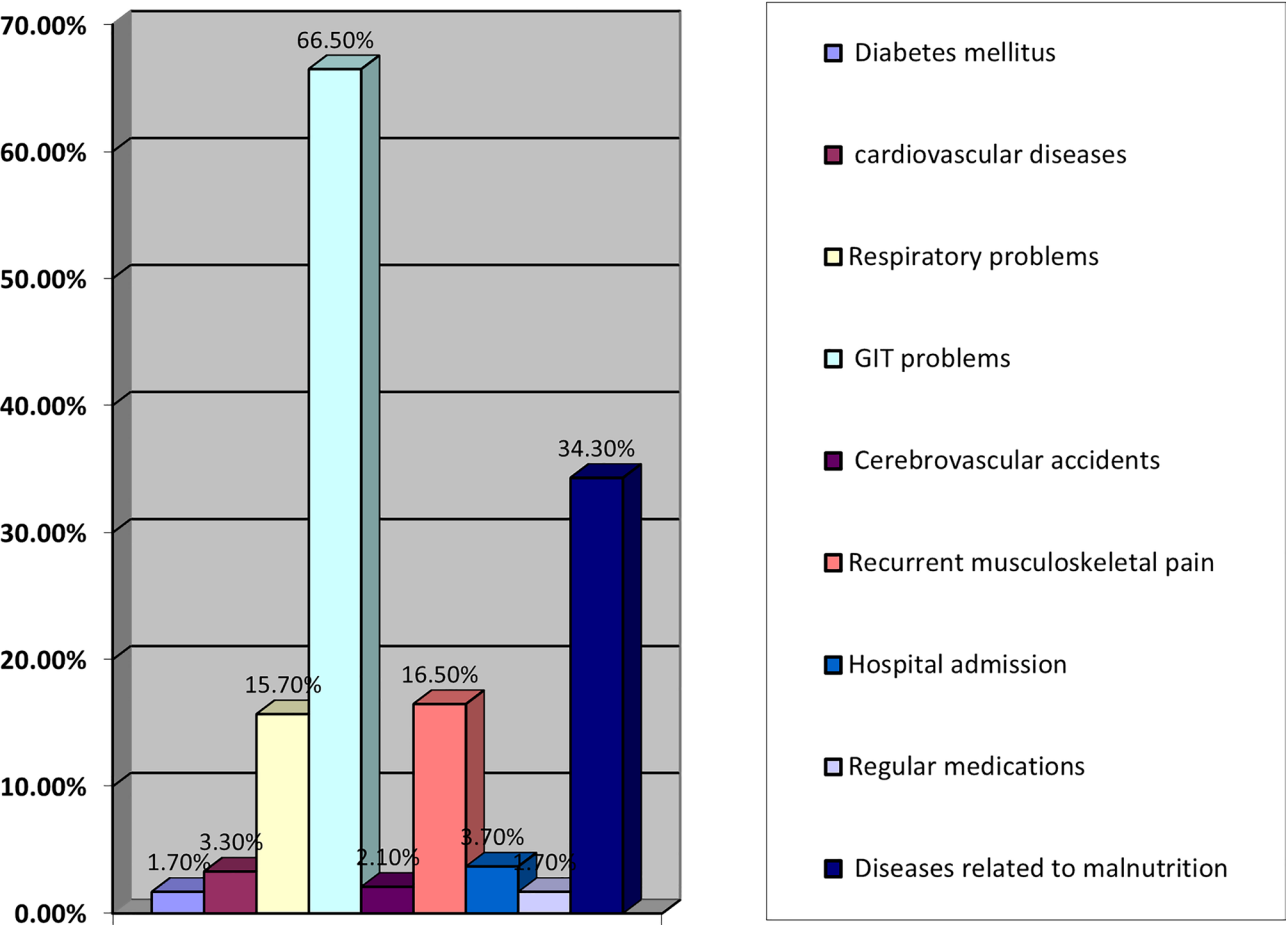
Physical health consequences

Table 2 highlights the prevalence of social factors among participants. Notably, 16% reported smoking, while none admitted to drug or alcohol use. Legal involvement was minimal, with only 1.2% of participants reporting such issues. Data presented in Tables 3 and 4, along with Figs. 1 and 2, reveal a concerning prevalence of psycho-behavioral and physical health challenges among the study participants with self-reported histories of childhood maltreatment.

**Table 1:** Sociodemographic characteristics of participants with histories of childhood maltreatment (*N* = 242)

Characteristic	Value
Age (years), mean ± SD	20.07 ± 1.14
Sex	
Male	91 (38.1%)
Female	151 (61.9%)
Residence	
Urban	202 (83.5%)
Rural	40 (16.5%)

**Table 2:** Distribution of long-term social consequences of childhood maltreatment among participants (*N* = 242)

Social factor	Yes, *n* (%)	No, *n* (%)
Smoking	16 (6.6%)	226 (93.4%)
Drugs/alcohol abuse	0 (0.0%)	242 (100.0%)
Involvement in legal issues	3 (1.2%)	239 (98.8%)

Note. Values are presented as frequencies and percentages of the total sample (*N* = 242)

**Table 3:** Distribution of long term psycho-behavioural consequences of child abuse among participants (*N* = 242)

Item	Yes, *n* (%)	No, *n* (%)
You usually describe your childhood as unhappy	63 (26.0)	179 (74.0)
Are you generally unsatisfied with yourself	87 (36.0)	155 (64.0)
Do you sometimes think that you are not good at all (low self-esteem)	168 (69.4)	74 (30.6)
Do you recurrently feel useless	190 (78.5)	52 (21.5)
Have you ever sought a psychiatric consultation	22 (9.1)	220 (90.9)
Do you feel generally anxious	182 (75.2)	60 (24.8)
Have you ever suffered from post-traumatic stress symptoms	126 (52.1)	116 (47.9)
Do you suffer from eating-related difficulties	115 (47.5)	127 (52.5)
Do you suffer from obsessive–compulsive symptoms	55 (22.7)	187 (77.3)
Do you suffer from behavioral difficulties (e.g., ODD-, CD-, ADHD-related symptoms)	45 (18.6)	197 (81.4)
Do you suffer from late comprehension	67 (27.7)	175 (72.3)
Have you ever suffered from educational difficulties	79 (32.6)	163 (67.4)
Do you have any activities that people may consider odd	54 (22.3)	188 (77.7)
Do you suffer from acute stress symptoms	62 (25.6)	180 (74.4)
Have you had the sense that you have a fatal disease	5 (2.1)	237 (97.9)

Note. Values are presented as frequencies and percentages of the total sample (*N* = 242)

**Table 4:** Distribution of long-term physical health consequences of child abuse among participants (*N* = 242)

Item	Yes, *n* (%)	No, *n* (%)
Do you suffer from diabetes mellitus	4 (1.6)	238 (98.3)
Do you suffer from cardiovascular diseases	8 (3.3)	234 (96.7)
Do you suffer from lung problems	38 (15.7)	204 (84.3)
Do you suffer from gastrointestinal problems	161 (66.5)	81 (33.5)
Have you had cerebrovascular accidents	5 (2.1)	237 (97.9)
Do you suffer from recurrent musculoskeletal pain	40 (16.5)	202 (83.5)
Have you been admitted to a hospital	9 (3.7)	233 (96.3)
Do you take regular medications	4 (1.7)	238 (98.3)
Do you have any diseases related to malnutrition (e.g., anaemia)	83 (34.3)	159 (65.7)

Note. Values are presented as frequencies and percentages of the total sample (*N* = 242)

### Psycho-behavioural Outcomes

A proportion of participants reported negative experiences, including feelings of unhappiness (26%), self-dissatisfaction (36%), inadequacy (69.4%), and recurrent uselessness (78.5%). Anxiety was also prevalent (75.2%). Notably, a substantial percentage of participants reported symptoms consistent with eating-related difficulties (47.5%), post-traumatic stress symptoms (52.1%), obsessive-compulsive symptoms (22.7%), and behavioral difficulties (81.4%). Regarding mental health care utilization, 22 participants (9.1%) reported having sought psychiatric consultation, while the majority (90.9%) reported no prior psychiatric consultation, Table 3; Fig. 1.

### Physical health outcomes

Gastrointestinal problems were the most frequent physical health concern, affecting 66.5% of participants. Other notable conditions included musculoskeletal pain (16.5%), lung problems (15.7%), and malnutrition-related diseases (34.3%). The prevalence of other conditions, such as diabetes (1.7%) and cardiovascular diseases (3.3%), was lower, Table 4; Fig. 2.

Overall, these findings describe a substantial burden of self-reported psycho-behavioral and physical complaints among participants with self-reported histories of childhood maltreatment.

## Discussion

### Psychological impact

This study provides descriptive data on self-reported psychological and physical difficulties among university students with self-reported histories of childhood maltreatment. The most frequently reported psycho-behavioral concerns included anxiety, feelings of inadequacy, low self-esteem, and trauma-related symptoms, while gastrointestinal complaints were the most frequently reported physical health complaint. Because the study was cross-sectional, descriptive, based on retrospective self-report, and conducted without a comparison group, the findings should be interpreted cautiously and should not be taken as evidence of causal, comparative, or independent relationships.

There are multiple factors that explain the associations discussed above. Previous studies have shown that exposure to childhood maltreatment is associated with the continual stimulation of the hypothalamic-pituitary-adrenal (HPA) axis. This has been interpreted to mean that those who experience this type of maltreatment have an increased likelihood of developing anxiety, emotional instability, and trauma-related symptoms as an adult.

However, direct comparisons should be interpreted with caution, as many prior studies relied on clinically diagnosed samples or standardized diagnostic interviews, whereas the present study assessed self-reported symptoms and perceived conditions within a non-clinical university population.

The present findings are broadly consistent with prior literature reporting psychological difficulties among individuals with histories of childhood maltreatment, although direct comparison should be made cautiously because the present study used descriptive self-report data in a non-clinical university sample [11]. Both studies demonstrate a clear pattern between childhood maltreatment and internalizing problems (e.g., anxiety, withdrawal), externalizing problems (e.g., aggression, hyperactivity), and depression. Differences in prevalence and help-seeking patterns across studies should be interpreted cautiously, as many prior investigations relied on clinically diagnosed samples using structured diagnostic interviews, whereas the present study captured self-reported psychological distress within a non-clinical university population. Notably, the 2023 study suggests that self-esteem may act as a protective factor against depression, particularly for male participants.

Differences in study populations may further explain variability in prevalence estimates, as several cited studies focused on clinical or high-risk samples, while the current investigation examined university students who may experience distress differently and may be less likely to seek formal psychiatric care.

Consistent with our findings, a prior study published in 2011 [12] documented a descriptive pattern between the type of childhood maltreatment and the severity of symptomology. The study revealed that emotional abuse and neglect independently contributed to greater functional impairment, while the co-occurrence of emotional abuse, emotional neglect, and physical abuse was associated with a diminished quality of life. While the 2011 study focused on clinically diagnosed individuals and assessed their symptoms while in treatment, this study provides additional support for the type of psychological distress caused by childhood maltreatment as being reported by individuals who do not have a formal psychiatric diagnosis and who are not receiving treatment for their emotional condition. Furthermore, the study noted a particularly detrimental effect of emotional abuse on social anxiety severity. However, this negative impact was demonstrably mitigated over time for participants who engaged in a specific intervention program.

A prior 2023 study reported a pattern between childhood maltreatment and multiple adverse outcomes in young adults [13]. These outcomes encompass heightened internalizing and externalizing psychopathology, compromised prefrontal cortex function, and overall lower psychological and functional well-being. Although the research referenced above used clinical samples, the present study adds descriptive self-report data from a university population reporting similar difficulties. The study also reported a potential relationship between childhood maltreatment and psychiatric disorders, particularly regarding post-traumatic stress–related symptoms. Individuals with a history of maltreatment and clinically identified or self-reported psychiatric symptoms exhibited a significantly higher prevalence of post-traumatic stress–related symptoms, comorbid psychiatric diagnoses, and earlier initiation of cannabis use. Furthermore, the study posits that the type and timing of childhood maltreatment experiences may influence the specific presentations of post-traumatic stress–related symptoms.

Prior literature has suggested that self-esteem may play a protective role; however, the present study was not designed to test buffering or mediating effects. Interventions that support self-esteem may be relevant for future research and student-support planning, but their effects were not evaluated in the present study. Such approaches may warrant consideration in future student-support research and service development.

The present findings are broadly consistent with prior literature reporting long-term consequences of childhood maltreatment, including those described in a recent investigation published in January 2023 [14]. The study demonstrated a compelling pattern between a history of childhood trauma and the emergence of high-risk behaviors in adolescents diagnosed with borderline personality disorder (BPD). It employed robust methods to elucidate clear patterns between various forms of childhood maltreatment and distinct high-risk behaviors within the BPD population. This finding is broadly consistent with existing literature on the potential influence of childhood trauma on BPD symptomatology. Among the different types of maltreatment explored, emotional abuse emerged as the most prominent factor reported in prior literature related to high-risk behaviors. Notably, the study suggests a gender-specific impact, with emotional abuse during childhood increasing the propensity for suicidal ideation in males and eating disorders in females. Moreover, the co-occurrence of emotional abuse and neglect in childhood was identified as the most significant risk factor associated with substance abuse. Prior literature highlights the potential relevance of early identification and intervention for childhood maltreatment. Recognition of specific forms of abuse and their potential long-term sequelae may help inform future targeted intervention planning to mitigate the development of high-risk behaviors in young people diagnosed with BPD.

The present findings are also broadly consistent with a study published in February 2022 [15]. This prior research identified significant patterns between family demographic characteristics, such as parental employment status and family composition, and the specific subtypes of childhood maltreatment experienced by individuals. Additionally, the study documented distinct behavioral patterns among children who had been subjected to physical abuse compared to those who had experienced emotional abuse, with neglect not exhibiting a differentiating effect. While the clinical generalizability of these findings may be limited, the research offers valuable insights for child welfare professionals. By understanding the potential variations in developmental trajectories associated with different forms of maltreatment, professionals can tailor interventions to individual needs. This personalized approach may be relevant to future efforts to reduce adverse long-term outcomes in children who have experienced maltreatment.

The present findings are also broadly consistent with prior literature describing adverse outcomes persisting into midlife, including those reported in a recent investigation [16]. This prior research employed robust methods to elucidate a significant pattern between a history of childhood maltreatment, encompassing both physical abuse and emotional neglect, and a multitude of adverse outcomes persisting into midlife. Specifically, the study identified that individuals with a documented history of childhood maltreatment were more likely to experience reduced educational attainment and cognitive functioning, financial hardship manifested as lower incomes and increased household expenditures, increased reliance on social welfare programs, greater susceptibility to chronic diseases and elevated rates of depression and economic hardship. Furthermore, the research revealed that childhood physical abuse emerged as a particularly potent predictor of difficulties with activities of daily living, unemployment, and reduced work hours. These findings in prior literature support the potential importance of early intervention strategies for childhood maltreatment. Such findings may help inform future prevention and intervention planning for maltreatment-related long-term outcomes.

The study found that only 9.1% of participants had sought psychiatric consultation even though many students reported experiencing high amounts of psychological distress and trauma-related symptoms. The lack of utilization of available mental healthcare services indicates a disparity between these two areas i.e. Psychological Distress and Seeking Psychiatric Consultation.

Several variables contribute to the low rates of help-seeking behaviour: Stigma around accessing mental health services, Limited Access to Specialized Services, and Normalisation of Distress (students may see their psychological symptoms as normal, typical and/or within their ability to cope). Some students may not consider it necessary to seek professional attention or feel they could not access such assistance.

Culture also plays a significant role in these areas. Most literature available regarding childhood trauma and mental health comes from Western countries: patterns of reporting of mental health care needs and accessing care vary by region or country. In Egypt stigma around both mental health and Childhood Abuse remain a significant barrier to open discussion and professional help; therefore, students may underreport their experience with trauma and/or may not follow through on their desire to seek assistance. To remedy these findings, we need to increase Mental Health Literacy, normalize help-seeking behaviour and increase accessibility to support services particularly in the university environment in which many students are struggling, silently.

### Physical health consequences

These descriptive findings are broadly consistent with prior literature reporting long-term physical and psychological difficulties among individuals with histories of childhood maltreatment, although the present study was not designed to test mechanisms or causal relationships. Experiencing chronic stress throughout childhood will have long-term effects on an individual’s body physiologically, including changes to neurobiological systems due to chronic activation and low-grade inflammation due to chronic stress, affecting the way an individual experiences pain, the function of their intestines, and their perception of physical symptoms. These changes could help explain why many people continue to experience physical symptoms even many years after experiencing the original stressors. The combination of the physiological effects of early life stress coupled with behaviors that are a result of early life stress (such as avoidance of the extensive use of the healthcare system or the lack of social support) can exacerbate the impact of stress over time. These two different types of pathways lead to the creation of physical health problems that continue to present challenges for people with a history of trauma as children.

Lending further credence to this finding, a separate investigation [17] documented a heightened prevalence of unexplained gastrointestinal symptomatology among adults with a history of childhood maltreatment. The study by Ringel et al. posits that this pattern may be mediated by long-lasting alterations in pain perception, a phenomenon known as nociception. Their research demonstrates that adult patients with unexplained abdominal pain who had experienced childhood abuse exhibited increased sensitivity to gut distention and altered patterns of central pain processing compared to controls without a history of maltreatment.

The current findings are broadly consistent with prior literature reporting long-term physical and psychological difficulties among individuals with histories of childhood maltreatment. These findings support the need for further research and may help inform future hypothesis generation and intervention planning.

### Practical implications

These descriptive findings suggest that universities may benefit from accessible mental health awareness, supportive referral pathways, and trauma-informed student services, particularly in contexts where stigma may limit help-seeking. Given the low proportion of participants who reported seeking psychiatric consultation despite substantial self-reported distress, universities may consider strengthening mental health literacy initiatives and improving access to supportive services for students who report psychological or physical difficulties. In addition, student support approaches should remain sensitive to the cultural and systemic barriers that may influence disclosure and help-seeking in settings such as Egypt and the broader MENA region. However, because the present study was descriptive, cross-sectional, and based on a non-validated self-report questionnaire without a comparison group, stronger evidence from studies using validated measures, comparison groups, and more analytically rigorous designs is needed before firm policy, screening, or intervention recommendations can be made.

### Limitations of the study

Researchers should be cautious when interpreting the results of this study because there were several limitations. First, the study was cross-sectional, making it difficult to definitively determine cause-and-effect relationships. Because the study was cross-sectional and descriptive, it cannot establish temporal or causal relationships between childhood maltreatment and later self-reported outcomes.

Childhood abuse is a sensitive issue and while some participants may have disclosed their experiences, others did not due to feelings of embarrassment or shame, personal coping strategies or cultural stigma. The effect of such factors may have resulted in lower reporting of childhood abuse among study participants. Therefore, it is possible that some of the participants were unable to recall their experiences with perfect accuracy or were influenced by time and/or pattern recognition. However, this does not nullify participants’ testimonies but rather demonstrates the need to be cautious in interpreting them.

The data were collected via questionnaires, which do not provide objective clinical assessments. Thus, although many participants self-reported experiencing distress and/or symptoms; the reported symptoms were purely subjective and unverified by a qualified professional. The differences in symptom report alone may explain why so few of the participants who reported emotional difficulties had received psychiatric treatment.

One limitation of this research is its lack of a confidence interval around its prevalence estimates, meaning we cannot tell how stable or accurate the prevalence values are. Additionally, while this survey was created specifically for the purpose of this study, it was not pilot tested or validated by subject matter experts, which impacts its ability to produce reliable assessment tools. Because the questionnaire was developed for this study and was not psychometrically validated, the findings should be interpreted as descriptive self-reported perceptions rather than standardized measurements of clinical outcomes.

We also need to consider that the cultural environment of Egypt affected how much and who chose to participate in the study. Its stigma regarding mental illness and child abuse continues to exist in Egypt, which would have impacted how many felt comfortable sharing their experiences or having access to mental health resources to do so. In addition to these variables, this study did not analyse differences based on sex or region or abuse type. The study did not measure or control potentially important confounders, including socioeconomic status, family context, current mental health status, and comorbid medical or psychiatric conditions. Because the study did not include a non-exposed comparison group, it cannot determine whether the observed prevalence of reported symptoms differs from that of university students without histories of childhood maltreatment. While these variances could provide very useful insights for future studies, this project aims to identify larger familial and community level trends and so will provide a foundation for expanded analyses in future studies that use larger, more representative samples.

## Conclusion

University students with a history of childhood maltreatment reported a high burden of self-perceived psychological distress and mental health–related symptoms, including anxiety, low self-esteem, feelings of inadequacy, and trauma-related symptoms, as well as physical health complaints, particularly gastrointestinal problems. Importantly, these findings reflect individuals’ self-reported perceptions of their mental and physical health rather than assessments conducted by trained professionals using standardized diagnostic procedures. These findings support the need for further research using validated tools and more rigorous designs, while also suggesting that universities may benefit from accessible mental health awareness and support pathways for students reporting distress.

## Electronic Supplementary Material

Below is the link to the electronic supplementary material.

Supplementary Material 1

## Data Availability

The availability of all the data and materials needed to complete the inquiry is attested to by the corresponding author.

## Abbreviations

BPD: Borderline personality disorder
