# Supplementary material for: Long term impact of child abuse in university students
Source: BMC Psychiatry. 2026 May 25;26:426. doi: 10.1186/s12888-026-08182-y (PMC13214125; doi:10.1186/s12888-026-08182-y)
Supplement: Supplementary file 1 — Supplementary Material 1 [file 12888_2026_8182_MOESM1_ESM.docx]

| Appendix (I) | | |
| --- | --- | --- |
| Psycho-behavioural consequences | | |
| Items | Yes | No |
| You usually describe your childhood as unhappy |  |  |
| Are you generally unsatisfied with yourself |  |  |
| Do you sometimes think that you are not good at all(low self-esteem) |  |  |
| Do you recurrently feel useless |  |  |
| Have you ever sought for a psychiatric consultation |  |  |
| Do you feel generally anxious |  |  |
| Have you ever suffered from post-traumatic stress disorder manifestations |  |  |
| Do you suffer from eating disorders |  |  |
| Do you suffer from Obsessive Compulsive Disorder |  |  |
| Do you suffer from behavioural disorders (as oppositional defiant disorder (ODD), conduct disorder (CD) and attention deficit hyperactivity disorder (ADHD)) |  |  |
| Do you suffer from late comprehension |  |  |
| Have you ever suffered from educational difficulties at any time |  |  |
| Have you any activities that people may consider odd |  |  |
| Do you suffer from acute stress disorder |  |  |
| Have you had the sense that you have a fatal disease |  |  |
| Physical health consequences | | |
| Items | Yes | No |
| Do you suffer from Diabetes mellitus |  |  |
| Do you suffer from cardiovascular diseases |  |  |
| Do you suffer from lung problems |  |  |
| Do you suffer from gastrointestinal problems |  |  |
| Have you had cerebrovascular accidents |  |  |
| Do you suffer from recurrent musculoskeletal pain |  |  |
| Have you been admitted in a hospital |  |  |
| Do you take regular medications |  |  |
| Do you have any diseases related to malnutrition (anaemia, for example) |  |  |
